# Regard for Law

**Published:** 1899-02

**Authors:** 


### Regard for Law.

There is quite noticeable a disposition in the dental profession to have a more strict observance of dental laws than has prevailed hitherto. We observe a number of States, among which is Iowa, California, Michigan, Missouri and several others, where dentists have been practicing, prosecuted, convicted and punished for violation of the dental law. Ostensibly these prosecutions have been for practicing without a license and without registration. The probability is, in most of these instances, a failure to register and receive a license was because of inability to pass examinations by Board of Examiners. It is to be hoped that every State in the Union having laws regulating the practice of dentistry will see to it that a full compliance with existing laws be observed by all practicing dentists. Dentists who take no interest in this question are not true to themselves, their profession nor to the public, and it is to be hoped that in every State the law will be strictly enforced.

There is no reason nor propriety in permitting a few incompetent and obstinate dentists to practice in violation of law, while a large majority are fully complying with its requirements. It is greatly to be desired that strict attention be given to this subject in every State in the Union.

